# Rapid Specific PCR Detection Based on *THCAS* and *CBDAS* for the Prediction of *Cannabis sativa* Chemotypes: Drug, Fiber, and Intermediate

**DOI:** 10.3390/ijms26115077

**Published:** 2025-05-24

**Authors:** Patwira Boonjing, Worakorn Wiwatcharakornkul, Chayapol Tungphatthong, Taksina Chuanasa, Somchai Keawwangchai, Tae-Jin Yang, Wanchai De-Eknamkul, Suchada Sukrong

**Affiliations:** 1 Center of Excellence in DNA Barcoding of Thai Medicinal Plants, Chulalongkorn University, Bangkok 10330, Thailand; patwira.b@chula.ac.th (P.B.); chubulaomega@gmail.com (C.T.); taksina.c@pharm.chula.ac.th (T.C.); 2 Department of Pharmacognosy and Pharmaceutical Botany, Faculty of Pharmaceutical Sciences, Chulalongkorn University, Bangkok 10330, Thailand; worakorn.ww@gmail.com; 3 Department of Chemistry, Faculty of Science, Mahasarakham University, Mahasarakham 44150, Thailand; somchai_2k@hotmail.com; 4 Department of Agriculture, Forestry and Bioresources, Plant Genomics & Breeding Institute, College of Agriculture & Life Sciences, Seoul National University, 1 Gwanak-ro, Gwanak-gu, Seoul 08826, Republic of Korea; tjyang@snu.ac.kr; 5 Natural Product Biotechnology Research Unit, Chulalongkorn University, Bangkok 10330, Thailand

**Keywords:** *Cannabis sativa*, THC, CBD, *THCAS*, *CBDAS*, drug-type, fiber-type, intermediate-type

## Abstract

*Cannabis sativa* L. is divided into three main groups: drug-type, intermediate-type, and fiber-type. The presence of tetrahydrocannabinol (THC) exceeding 0.2–0.3% in drug-type and intermediate *Cannabis* that utilized for recreational and medicinal purposes renders them illegal due to potential mental health implications. Fiber-type contains high cannabidiol (CBD) and low THC, making it suitable for household use such as textiles and animal feed. Accurate classification is essential to prevent misuse of the plant. High-performance thin-layer chromatography (HPTLC) and ultra-performance liquid chromatography (UPLC), used respectively for the qualitative and quantitative analyses of THC and CBD particularly in female inflorescences, categorized 85 samples of 46 cultivars used in this study into three distinct chemotypes. While chemotype analysis of a very specific organ of the plants accurately identifies *Cannabis* groups, it requires time-consuming plant development to maturity. Genotype analysis targeting tetrahydrocannabinolic acid synthase (*THCAS*) and cannabidiolic acid synthase (*CBDAS*) genes offers a faster alternative for classifying *Cannabis* types, allowing for sample determination from any part at any developmental stage of the plant. DNA sequencing allowed a phylogenetic analysis based on these genes, classifying all 85 samples of 46 cultivars into the same three groups identified by chemotype analysis. This study is the first to successfully examine the relationship between chemotype and genotype in 85 samples of 46 cultivars. Rapid identification of *Cannabis* types through genotype analysis lays the groundwork for future development of detection kits.

## 1. Introduction

*Cannabis* (*Cannabis sativa* L.) belongs to the Cannabaceae family [1], and has been utilized by humans for medicinal, culinary, and fiber purposes for a significant period [2]. Several countries have excluded *Cannabis* from their lists of narcotic plants under the *Cannabis* Act and the Food and Drug Act. According to [3], *Cannabis* is classified into three primary types based on its usage: drug-type containing 1–20% tetrahydrocannabinol (THC) using for reducing chronic pain [4], alcoholism and drug addiction [5], depression [6], and epilepsy [7]; fiber-type with high cannabidiol (CBD) and THC content below 0.2% or 0.3% depending on *Cannabis* law of each country using for refining into paper, rope, textiles, biofuel, food, and animal feed [8]; and intermediate-type with a THC:CBD ratio of 1:1 using for reducing parkinson’s disease [9] and multiple sclerosis [10]. The cultivation of fiber-type cultivars is permitted without prior authorization, as long as the THC content remains below the legal threshold [11]. However, there is a widespread issue of misidentified *Cannabis* seeds being sold in markets, for example, drug-type *Cannabis* seeds are adulterated with the fiber-type seeds leading to the unintentional cultivation of illegal narcotic plants in some cases. Therefore, accurate identification of the *Cannabis* chemotype is crucial to ensure its appropriate use.

There are primary methods for identifying plant species, including *Cannabis*: morphology, chemotyping, and genotyping. The morphological method, based on leaflet characteristics, achieved a 92.9% accuracy rate compared to chemotyping. However, this method is time-consuming and requires a high level of taxonomy identification skills [12]. The chemotype method, widely used, divides *Cannabis* into three types: chemotype I (drug-type), chemotype II (intermediate-type), and chemotype III (fiber-type), characterized by THC/CBD ratios greater than 1, nearly equal to 1, and less than 1 [3,13]. Recently, chemotype classification has been based on histogram frequency distributions of log10 THC/CBD [14,15]. Although HPLC has been the primary method for chemotype identification, it requires significant solvent and time consumption, making it impractical for rapid identification of forensic samples in real-time scenarios. High-Performance Thin-Layer Chromatography (HPTLC) offers a faster alternative capable of simultaneously analyzing a large number of samples. It can autonomously spot up to 75 samples on up to five HPTLC plates.

One of the limitations of chemotype classification is that cannabinoids including THC and CBD can be identified at high concentrations in mature female inflorescence tissue [16,17]. These major cannabinoids are synthesized and accumulated in glandular trichomes on the aerial parts of the plant during the 6 to 7 weeks after planting [18]. This poses a challenge because *Cannabis* seeds are often adulterated, and chemotype detection requires a long growth period until female inflorescences emerge. As DNA is consistent throughout the plant, DNA markers have been developed to be an alternative method. These markers are divided into two types including DNA barcode and taxon-specific markers. DNA barcode markers use universal primers targeting highly conserved sequences such as chloroplast DNA and nuclear DNA [19]. However, these markers have low discrimination power and amplification issues in some species. For example, nuclear DNA (ITS) and chloroplast DNA (trnH-psbA intergenic spacer and matK) are unable to discriminate between wild potatoes species [20]. Taxon-specific markers, such as Single Nucleotide Polymorphisms (SNPs), offer better classification from species to variety levels due to marker presence-absence variance. In hop (*Humulus lupulus*), closely related to *Cannabis*, seven SNP markers successfully discriminated 116 distinct hop varieties [21]. In *Cannabis*, tetrahydrocannabinolic acid synthase (*THCAS*) and cannabidiolic acid synthase (*CBDAS*) genes are used to develop SNP markers to classify drug-type and fiber-type [22,23,24,25]. *THCAS* and *CBDAS* follow a multi-locus model connected by genome-wide analysis [26]. Drug-type is reported to be *THCAS* active and *CBDAS* inactive, while fiber-type is *CBDAS* active but *THCAS* hypoactive, with poor enzymatic function [24]. Therefore, classification using both chemotype and genotype is more recommended for its convenience and time efficiency [27].

In a previous study, the relationship between chemotype and genotype in *Cannabis* was investigated using HPLC and DNA markers for *THCAS* and *CBDAS*. For genotyping, the B1192 and D589 markers were utilized for *THCAS*, while the B1080 marker was used for *CBDAS*. The results indicated that the B1192 and B1080 markers achieved an accuracy of 19.23%, correctly identifying only intermediate-type and fiber-type. The D589 marker showed 100% accuracy but was only able to detect *THCAS* [28]. Moreover, unlike the three intermediate-type cultivar samples from previous studies that lacked DNA sequencing, our study includes 11 sequenced intermediate-type samples. However, no studies to date have successfully conducted both chemical and genetic analyses of *Cannabis* plants within a single investigation. Thus, our study aims to rapidly classify *Cannabis* 3 types including drug-type, intermediate-type, and fiber-type by determining THC and CBD contents using HPTLC, UPLC, along with developing *THCAS* and *CBDAS* DNA markers using SNPs at the 3′ end of primers. These methods are expected to help farmers and entrepreneurs in Thailand by reducing cultivation costs and accurately identifying *Cannabis* chemotypes, thereby eliminating the need to grow *Cannabis* to maturity for its female inflorescences.

## 2. Results

### 2.1. HPTLC Chemical Profile, UPLC Content Analysis and Heatmap Clustering Analysis

**Figure 1:**
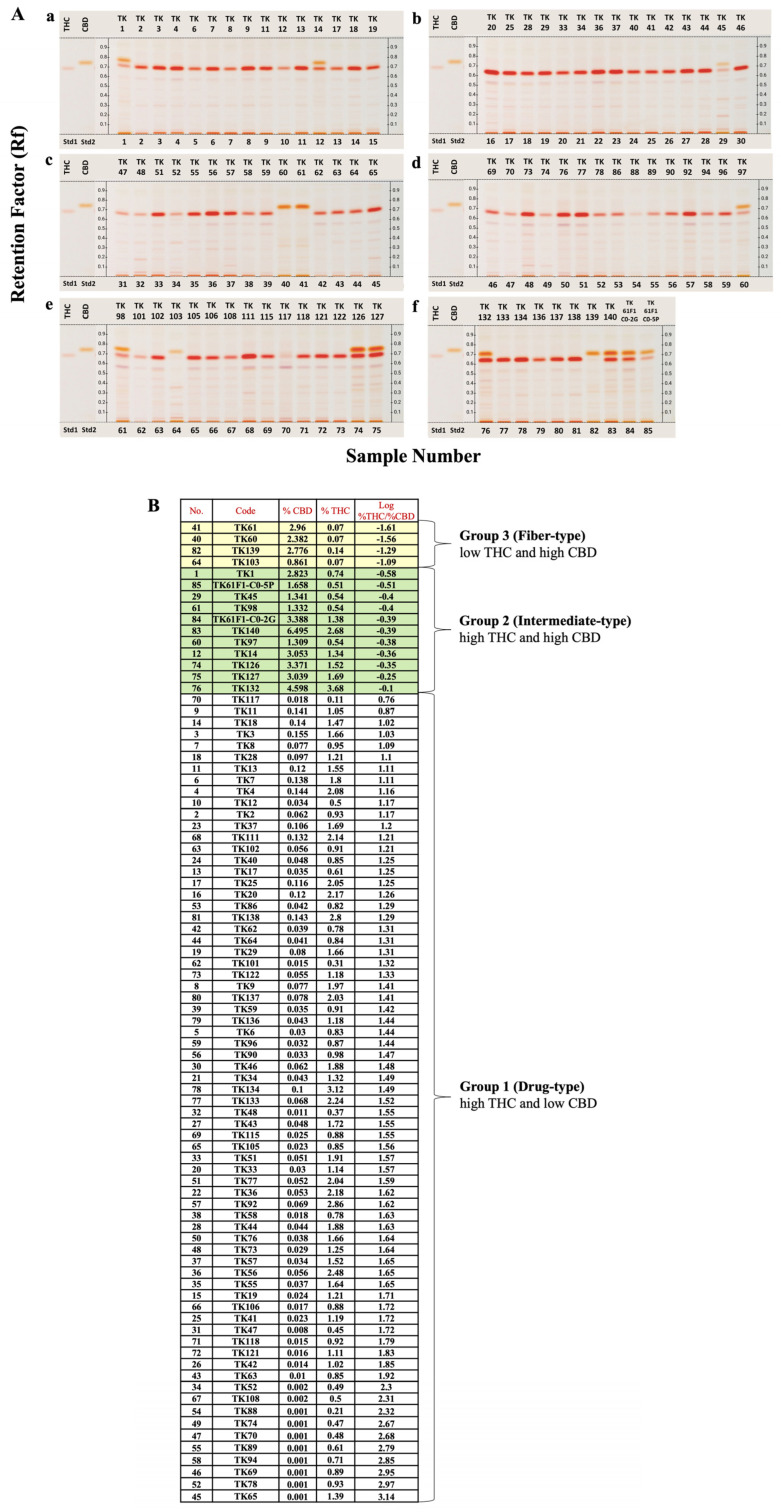
(**A**) HPTLC chemical profile of *Cannabis* 85 cultivars on 6% diethylamine in toluene mobile system. *Y*-axis represented R_F_ value. Standards THC and CBD are shown on the left-hand side of each plate. Lanes 1 to 85 are *Cannabis* samples (TK1 to TK61F1-CO-5P);(a) Lanes 1 to 15 (TK1 to TK19); (b) Lanes 16 to 30 (TK20 to TK46); (c) Lanes 31 to 45 (TK47 to TK65); (d) Lanes 46-60 (TK69 to TK97); (e) Lanes 61 to 75 (TK98 to TK127); (f) Lanes 76-85 (TK132 to TK61F1-CO-5P). Application volume for all lanes is 3 μL. (**B**) Calculated THC and CBD contents and their %THC/%CBD ratios based on UPLC analysis.

**Figure 2:**
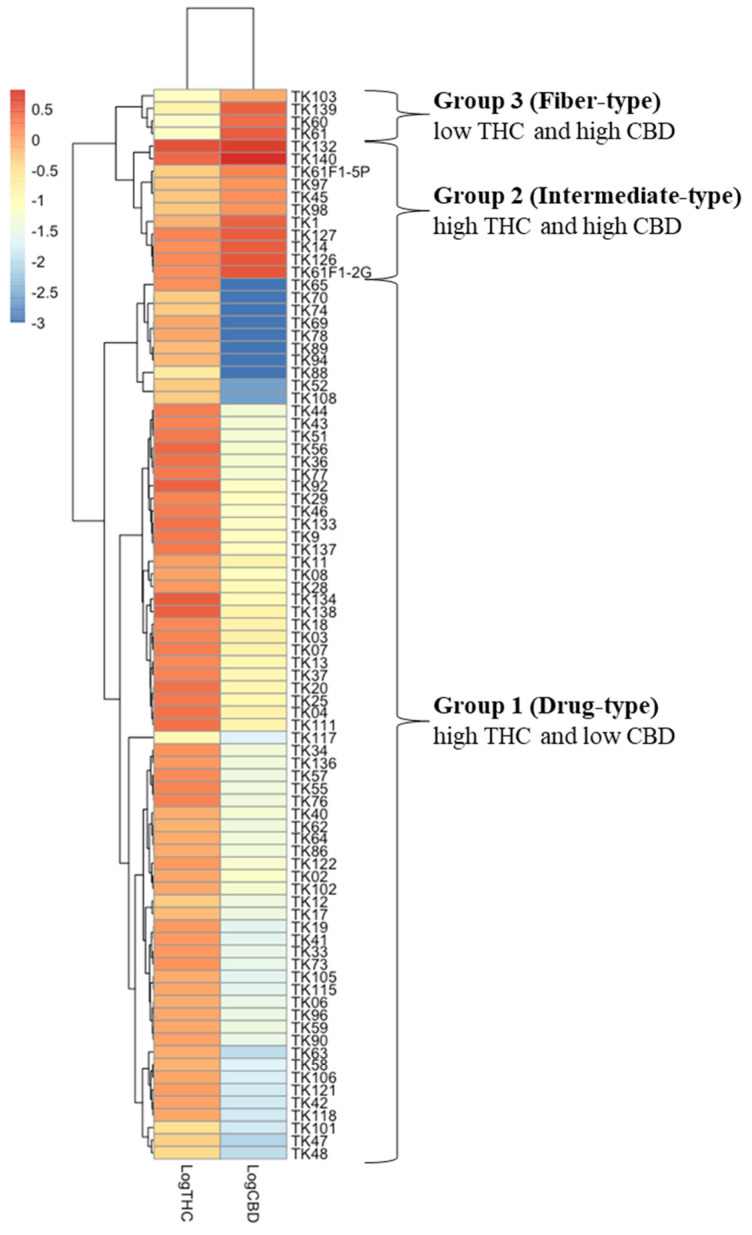
Heat maps analysis of chemical constituents for *Cannabis* plants grouping by using neutral form including THC and CBD, made with RStudio (Version 1.4.1103). To classify *Cannabis* group by quantitative analysis of UPLC, THC and CBD contents were log10 transformed and are displayed as colours ranging from red to blue as shown in the key. Both rows and columns are clustered using the Euclidean clustering metric and Complete method of the pheatmap package.

The analyses of chemical profiles and THC and CBD contents from 85 samples of 46 *C.sativa* cultivars were performed using HPTLC and UPLC, respectively. The resulting chemical profiles revealed three distinct patterns: drug-type (chemotype I), intermediate-type (chemotype II) and fiber-type (chemotype III). As shown in Figure 1A, the majority of the samples appeared to be the drug-type characterized by high THC and low CBD contents, followed by the intermediate-type with approximately 1:1 THC/CBD ratio in 11 samples of TK1, TK14, TK45, TK97, TK98, TK126, TK127, TK132, TK140, TK61F1-C0-2G, and TK61F1-C0-5P, and the fiber-type marked by high CBD and low THC contents in four samples of TK60, TK61, TK103, and TK139. UPLC chromatograms and peak areas for all samples were meticulously determined, and standard curves for THC and CBD were used to calculate their contents from the peak areas (Figure S1). The resulting THC and CBD contents, along with their log %THC/%CBD ratio values, are summarized in Figure 1B. These %THC and %CBD values were subsequently used in a heatmap clustering analysis, displayed in varying shades, which grouped the samples into three distinct clusters (Figure 2). The heatmap results were consistent with the HPTLC chemical profile, supporting the classification of *Cannabis* samples based on log %THC/%CBD ratios and showing a clear separation into three groups without outliers. According to the heatmap clustering, plants with log %THC/%CBD ratios ranging from 0.76 to 2.31, −0.58 to −0.10, and −1.61 to −1.09 were classified as drug-type, intermediate-type, and fiber-type, respectively.

### 2.2. A Phylogenetic Study by DNA and Amino Acid Sequence Analysis

**Figure 3:**
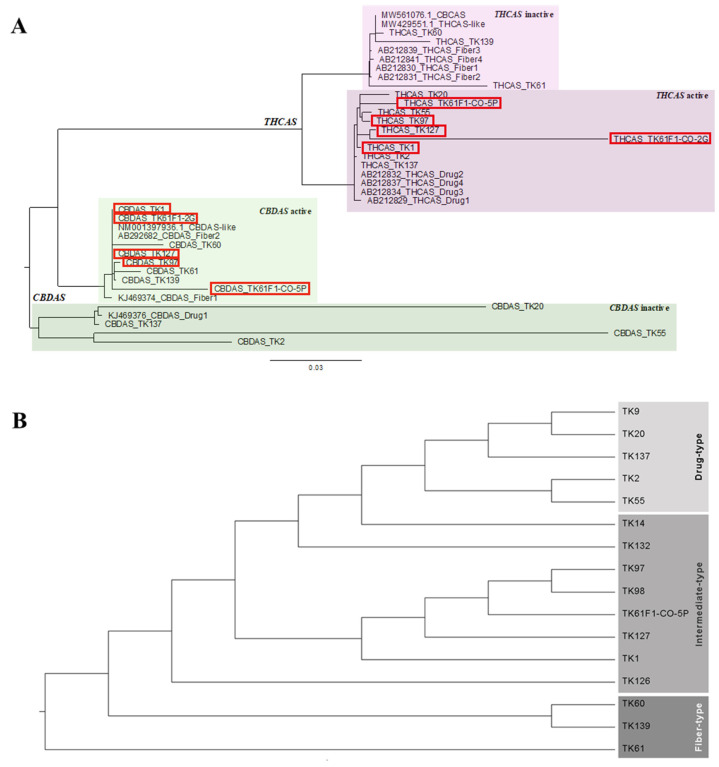
(**A**) Phylogenetic tree of *THCAS* and *CBDAS* genes of *Cannabis* samples including drug-type (TK2, TK20, TK55, and TK137), intermediate-type (TK1, TK97, TK127, TK61F1-CO-2G, and TK61F1-CO-5P), and fiber-type (TK60, TK61, and TK139). Reference sequences were used including *THCAS* drug-type (AB212829, AB212832, AB212834, and AB212837), *THCAS* fiber-type (AB212830, AB212831, AB212839, and AB212841), *THCAS*-like (MW429551.1), *CBCAS* (MW561076.1), *CBDAS* drug-type (KJ469376), *CBDAS* fiber-type (KJ469374 and AB292682), and *CBDAS*-like (NM001397936.1). The red box indicated intermediate-type samples. (**B**) Phylogenetic tree of combined *THCAS* and *CBDAS* genes of *Cannabis* samples including drug-type (TK2, TK9, TK20, TK55, and TK137), intermediate-type (TK1, TK14, TK97, TK98, TK126, TK127, TK132, and TK61F1-CO-5P), and fiber-type (TK60, TK61, and TK139). Reference sequences were excluded as they are from different sources and cannot be combined together.

The samples selected for phylogenetic analysis exhibited clear bands of CBD- and/or THC-related metabolites and clear *THCAS* and *CBDAS* DNA sequencing results. These representative samples were based on chemotype including four drug-type amples (TK2, TK20, TK55, and TK137), five intermediate-type samples (TK1, TK97, TK127, TK61F1-C0-2G, and TK61F1-C0-5P), and three fiber-type samples (TK60, TK61, and TK139). A phylogenetic tree was constructed to investigate the evolutionary relationships between *THCAS* and *CBDAS* genes in *Cannabis* (Figure 3A). The analysis utilized a reference database of *Cannabis THCAS* and *CBDAS* sequences (Table S1). The tree revealed four main groups: active *CBDAS* (fiber-type *CBDAS* references, *CBDAS*-like reference, and intermediate-type and fiber-type *CBDAS* samples), inactive *CBDAS* (drug-type *CBDAS* references and drug-type *CBDAS* samples), active *THCAS* (drug-type *THCAS* references and drug-type and intermediate-type *THCAS* samples), and inactive *THCAS* (fiber-type *THCAS* references, *THCAS*-like reference, *CBCAS* reference, and fiber-type *THCAS* samples). The *CBCAS* reference sequence was included in this phylogenetic tree study due to its high sequence similarity to *THCAS* and their overlapping biochemical activity. Both enzymes, *THCAS* and *CBCAS*, catalyze reactions involving the same precursor molecule, cannabigerolic acid (CBGA). According to phylogenetic analyses from [29], they are classified into distinct subclades: *THCAS* in subclade A1 and CBCAS in subclade A2. In cannabis plants lacking a functional *THCAS* gene, certain *CBCAS* variants may compensate by producing trace amounts of THCA, thereby influencing the plant’s chemotype. This observation suggests a degree of functional redundancy or enzymatic flexibility within the cannabinoid synthase gene family [30]. The phylogenetic analysis revealed that intermediate-type cannabis possessed a *THCAS* gene sequence similar to that of drug-type *Cannabis* and a *CBDAS* gene sequence similar to that of fiber-type *Cannabis*. To distinguish the intermediate-type from drug-type and fiber-type samples, a phylogenetic tree was constructed using combined *THCAS* and *CBDAS* gene sequences. The results showed that intermediate-type samples clustered between the drug-type and fiber-type groups, confirming our hypothesis (Figure 3B). In addition, *THCAS* was fully translated into full-length amino acid sequences in drug-type and intermediate-type samples, while it was encoded as truncated proteins in fiber-type samples (Figure S2A). Conversely, *CBDAS* was translated into partial amino acid sequences in drug-type samples, whereas intermediate-type and fiber-type samples processed full-length amino acid sequences encoded from this gene (Figure S2B). Consequently, DNA markers targeting *THCAS* drug-type and *CBDAS* fiber-type were designed based on the phylogenetic tree.

### 2.3. Development of DNA Markers Based on THCAS and CBDAS

**Figure 4:**
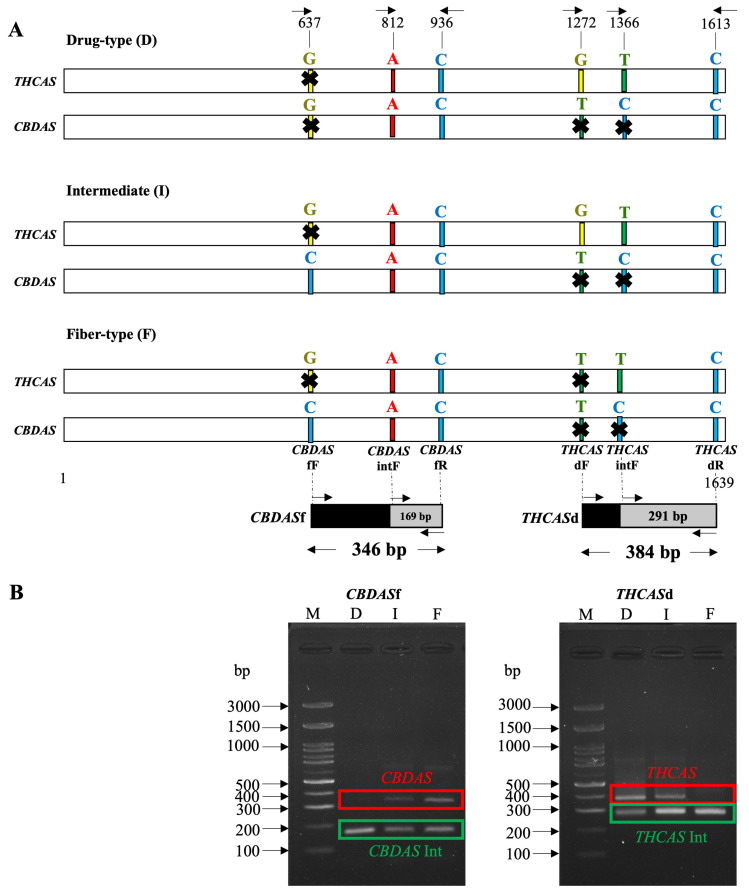
(**A**) *THCAS* and *CBDAS* specific primers at 3′ position for *Cannabis* types for prediction including Drug-type, Intermediate, and Fiber-type. The forward and backward arrows indicate the directions of the forward and reverse primers, respectively. (**B**) Expected PCR products (red box indicated) for *THCAS*d (384 bp) and *CBDAS*f (346 bp) with their internal controls (green box indicated) including *THCAS*int (291 bp) and *CBDAS*int (169 bp). The arrows indicate DNA bands of defined base pair sizes in the standard DNA ladder (lane M).

DNA markers for the separation of the three types of *Cannabis* were developed, targeting the active cannabinoid synthase genes, including *THCAS* for drug-type and intermediate *Cannabis* and *CBDAS* for fiber-type and intermediate *Cannabis*. Single nucleotide polymorphisms at the 3′ end of designed forward primers were used to differentiate between each *Cannabis* type (Figure 4A). The expected PCR product diagrams for each DNA marker were presented (Figure 4B). For *THCAS* drug-type and intermediate-type detection using primer pairs D (Table S2), no band was detected in TK60, TK61, TK103, and TK139 (Figure S3A). All non-detected samples were fiber-type. On the other hand, the target band using primer pairs F for *CBDAS* fiber-type and intermediate-type (Table S2) was amplified in all fiber-type and intermediate samples including TK1, TK14, TK45, TK60, TK61, TK97, TK98, TK103, TK126, TK127, TK132, TK139, TK140, TK61F1-CO-2G, and TK61F1-CO-5P (Figure S3B). An internal control for *THCAS* and *CBDAS* was detected in every sample except TK61, which showed no band for the *THCAS* internal control using primer pairs C. To support the reliability of this method, sensitivity and specificity tests were conducted. DNA was extracted from three representative cultivars—TK19 (Drug-type), TK97 (Intermediate-type), and TK60 (Fiber-type)—and diluted from 10 µg/mL to 0.625 µg/mL (Figure S4A). At concentrations of 10 µg/mL and 5 µg/mL, all chemotypes were successfully detected using both *THCAS* and *CBDAS* markers. Error rates observed at lower concentrations—2.5 µg/mL, 1.25 µg/mL, and 0.625 µg/mL—were 16.67%, 50%, and 66.67%, respectively. Furthermore, visual inspection of PCR product bands revealed a strong correlation between band intensity and DNA concentration. In most cases, clearer and more distinct bands were observed at higher DNA concentrations, while faint or absent bands were seen at lower concentrations. This trend underscores the impact of DNA input quantity on amplification efficiency and final PCR product visibility. These results demonstrate that 5 µg/mL is the minimum recommended DNA concentration to ensure consistent, accurate, and visibly detectable PCR results across all Cannabis chemotypes. To evaluate specificity, the designed markers were tested on both Cannabis tissues and closely related or unrelated plant species. A blind test on an unknown Cannabis seed correctly identified it as Drug-type, based on the exclusive presence of the *THCAS* marker. No amplification signals were detected in *Humulus lupulus* (hop), a close relative from the Cannabaceae family, or in unrelated Zingiberaceae species (*Amomum biflorum* and *Zingiber montanum*), confirming the high specificity of the primers (Figure S4B). These findings validate that our PCR-based detection system is both highly specific and sufficiently sensitive for practical application.

### 2.4. The Relationship Between Chemotype and Genotype

The log %THC/%CBD ratios calculated in Figure 1B, which correlated with both the HPTLC chemical profile patterns and heatmap analysis, revealed three distinct groups. Plants with log %THC/%CBD ratios ranging from 0.76 to 2.31, −0.58 to −0.10, and −1.61 to −1.09 were classified as drug-type, intermediate-type, and fiber-type, respectively. For genotype prediction, *THCAS* primer band presence in drug-type and intermediate-type targets was represented with T, while its absence in fiber-type was represented with t. *CBDAS* primer band presence in fiber-type and intermediate targets was represented with D, while its absence in drug-type was represented with d. The total genotype was determined from the results of using two pairs of primers, one for *THCAS* and one for *CBDAS*. There were three patterns of total genotype (TD, Td, and tD), leading to three different results: drug-type (Td), intermediate (TD), and fiber-type (tD) (Table 1). The accuracy percentage of genotype and chemotype matching was 100% (85/85).

## 3. Discussion

This is the first report that used 85 samples of 46 *Cannabis* cultivars from different regions of origin all around the world (Figure S6; Table S1) However, group classification by HPTLC chemical profile was challenging due to the high number of parameters that needed proper adjustment [31]. Therefore, for accuracy, UPLC was used for the quantitative analysis of in the *Cannabis* samples. THC and CBD, which are major and stable compounds, were the only ones analyzed for *Cannabis* grouping by heat map (Figure 2). The result correlated with the HPTLC profile pattern, which separated into three groups: Group 1 with high THC, Group 2 with mixed moderate content of THC and CBD, and Group 3 with high CBD, classified into drug-type, intermediate-type, and fiber-type, respectively [13]. Several studies have simplified *Cannabis* chemotype classification by analyzing histogram frequency distributions of the log %THC/%CBD ratio, using threshold values of 0.0 [32] and −1.0 as arbitrary cutoffs to differentiate between chemotypes I and II [14,33]. Most of the 85 samples used in this study were classified as drug-type, originating from *Cannabis* seeds sent to Dr. Somchai Keawwangchai at Mahasarakham University. These seeds were collected from various regions across Thailand, presumably by individuals who cultivated and consumed *Cannabis* for health-related or recreational purposes. As these strains typically contain THC, the majority of the samples fell into Group 1 (drug-type). An outlier case was observed in the TK117 sample, which showed both low THC levels—comparable to those of fiber-type samples—and low CBD levels based on HPTLC profiling. However, its overall chemical profile closely resembled that of the TK88 sample. Notably, TK88 was classified as a high-THC sample, ranking 78th out of 85 based on the log(%THC/%CBD) ratio. Given the similarity in their profiles, TK117 would be expected to cluster with TK88 in Figure 2. It is possible that a technical error occurred during the CBD content analysis of TK117 using UPLC. This is supported by the high variability observed in the three technical replicates for CBD quantification (mean: 0.018; SD: 0.014). The inconsistency may be attributed to the sensitivity limitations of the UPLC method, as CBD bands were not visible in any of the drug-type samples, suggesting very low CBD concentrations. We plan to further investigate the limitations of the UPLC method for detecting CBD in these samples in future work. We also performed the HPTLC experiment on our *Cannabis* samples after two years of storage and found that THC had degraded into CBN, resulting in chemotype misidentification (Figure S5). Ref. [34] reported that environmental factors such as herbivory, excessive heat, and drought can significantly affect cannabinoid production in two-week-old *Cannabis* plants. In particular, exposure to a seven-day drought caused a marked reduction in both THC and CBD levels. To minimize such environmental effects in our study, all *Cannabis* samples were grown indoors under controlled conditions, including a constant temperature of 25 °C, 50–60% humidity, and a 12-h light/dark cycle.

To find the relationship between Chemotype and Genotype. DNA sequencing of *THCAS* and *CBDAS* of 3 types of *Cannabis* was analyzed. The results showed four clearly separated groups (Figure 3A). Group 1 showed a group of *CBDAS* inactive with all drug-type samples [35,36]. A previous report identified a 4 or 6 bp frame-shift deletion at position 153, causing *CBDAS* mutation to be inactive [28]. Group 2 indicated *CBDAS* active including *CBDAS* fiber-type references and *CBDAS*-like sequences [29]. Samples in intermediate-type and fiber-type samples were grouped together because they both contain the active form of *CBDAS*, which is responsible for producing high levels of CBD. Group 3 showed *THCAS* active including *THCAS* drug-type references with high THC (drug-type) and high THC and CBD (intermediate-type) samples. *THCAS* has been functionally characterized [37], indicating its correlation with the drug-type chemotype. Interestingly, this study confirmed that intermediate-type samples were also in this group due to the high THC amount converted from the active form of the *THCAS* enzyme. Group 4 comprised *THCAS* inactive samples, which included *THCAS* fiber-type references along with our fiber-type samples, specifically TK60, TK61, and TK139. The inactivity of *THCAS* in this group is attributed to amino acid substitutions, which induce changes in enzymatic activity in *THCAS* fiber-type samples [22]. However, *THCAS* amino acid sequence of TK139 was fully translated showing active enzymatic activity (Figure S2A). Since it was not a complete *THCAS* due to sequencing limitation, stop codon was found only in TK139 *THCAS* sequence before amino acid translation spot. Therefore, *THCAS* enzymatic activity of TK139 was probably inactive. Additionally, the presence of *THCAS*-like (a nonfunctional pseudogene) and *CBCAS*, containing mutated *THCAS* sequences from drug-type cultivars [29,38], as well as fiber-type *THCAS* sequences [22], confirms *THCAS* inactivation in high CBD (fiber-type) samples. These findings align with prior research indicating the evolutionary relationship between *THCAS* and *CBDAS*, with *THCAS* originating from *CBDAS* [35].

However, this separated phylogenetic tree have a limitation to identify the intermediate-type plants, which often harbor active alleles of both *THCAS* and *CBDAS*, leading to ambiguity when the genes are analyzed in isolation. To overcome this, we employed a concatenated phylogenetic approach, in which *THCAS* and *CBDAS* sequences were combined into a single dataset for phylogenetic analysis. This method provided a clearer and more robust classification of the three *Cannabis* types, as demonstrated in our resulting tree. By increasing the total sequence length and combining genetic information across loci, the phylogenetic signal was significantly strengthened, allowing for more accurate clustering based on chemotypic characteristics. This strategy is consistent with findings from previous studies, which have demonstrated that concatenating multiple gene fragments enhances phylogenetic resolution, particularly in cases where single-gene analyses are insufficient. For instance, Ref. [39] emphasized that combining multiple gene fragments can uncover hidden phylogenetic signals and improve the accuracy of evolutionary inference compared to single-gene analysis. Similarly, a study on green algae within the class Zygnematophyceae by [40] demonstrated that combining *SSU rDNA* and *rbcL* gene sequences yielded a phylogeny that was not only more resolved but also more consistent with morphological classifications. In the animal kingdom, Ref. [41] applied a concatenated approach combining mitochondrial and nuclear DNA to accurately resolve species-level relationships in the mosquito genus *Anopheles*. Their work underscores the applicability of this method in complex evolutionary lineages and highlights its utility in confirming species identities. Taken together, our findings support the broader conclusion that concatenated gene analysis is a powerful tool in resolving complex phylogenetic relationships. In the case of *Cannabis*, it enables a more definitive classification of plant types.

DNA markers for *Cannabis* group classification were successfully developed based on SNPs of the active form of each enzyme, especially for the intermediate group, which contains both *THCAS* and *CBDAS* active forms. Specific primers were designed with a 3′ end different from the inactive form. While universal primers of *THCAS* (D589) and *CBDAS* (B1080/B1192) have been widely used for identifying *Cannabis* types, a challenge arises with B1080/B1192, which is a sequence-characterized amplified region (SCAR) marker requiring precise amplicon size detection. The minimal size differences of *THCAS* drug-type and *CBDAS* fiber-type (1192 and 1080 bp, respectively) increase the risk of misrecognition [28,42]. Our primers were designed for a PCR-based method similar to the D589 primer. Previous studies relied solely on *THCAS* drug-type specific primers with 1.2-kilobase amplicon sizes [22,23]. However, the PCR process was time-consuming. To address this, reducing the size of PCR products was recommended. *CBDAS* fiber-type specific primers were initially introduced by [24]. Nevertheless, the *CBDAS* primers could also amplify *THCAS* in drug-type *Cannabis*, resulting in nonspecific amplification. To overcome this issue, new active *THCAS* and *CBDAS* primers were designed and tested with drug-type and fiber-type *Cannabis* [25]. However, there was no intermediate-type sample to identify with these PCR primers. To address this gap, our study used 11 cultivars of intermediate *Cannabis* from Europe and Asia continents, demonstrating that this *Cannabis* type could indeed be identified using both active *THCAS* and *CBDAS* primers.

In conclusions, this study represents the first report to integrate data from chemotype and genotype analysis, enhancing the accuracy of predicting *Cannabis* chemotypes. Most previous studies in this area have focused primarily on the application of general SNP markers or non-functional gene regions for chemotype differentiation in *Cannabis sativa*. In contrast, our research emphasizes allele-specific SNP markers designed based on the functional regions of *THCAS* and *CBDAS* genes. These markers are highly correlated with the actual cannabinoid production phenotypes and allow for precise discrimination among drug-type, fiber-type, and intermediate-type cannabis. Additionally, our study integrates multiple *Cannabis* chemotypes and verifies genotypic classification through direct chemotype analysis using HPTLC and UPLC. This dual-validation approach strengthens the predictive power and reliability of our detection method. The ability to distinguish chemotypes accurately using DNA extracted from any part of the plant—including seeds and leaves—further enhances the practical utility of our method for breeding programs, quality control, and regulatory compliance. We anticipate that these features represent a significant advancement over existing methods, particularly in terms of accuracy, applicability, and field-level implementation. It is considerably interesting to develop a test kit for rapid *Cannabis* identification in the future.

## 4. Materials and Methods

### 4.1. Cannabis Samples Collection

The collection of *Cannabis* samples involved the gathering of dried female inflorescences and leaves from 85 samples of 46 *C. sativa* cultivars from different regions of Thailand and globally (Figure S6) including 51, 26, and 2 from Asia, Europe, and America continent, respectively. These samples, listed in Table S1, were provided by Somchai Keawwangchai. For chemical component analysis, 20 mg of inflorescences and leaves were utilized. All *Cannabis* samples were screened using HPTLC and quantified by UPLC. DNA marker prediction was performed on the same plant parts.

### 4.2. Chemical Component Analysis by HPTLC

#### 4.2.1. Chemicals and Reagents

Diethylamine (analytical grade) and toluene (analytical grade) were procured from Merck, Darmstadt, Germany and Honeywell, Offenbach, Germany, respectively. Analytical grade methanol was acquired from Merck, Darmstadt, Germany. Mobile phases, comprising diethylamine and toluene, were prepared using the ratios 6:94. Fast Blue B salt, obtained from Glentham Life Sciences, Corsham, UK, was used for staining purposes.

#### 4.2.2. Cannabinoid Standards

Certified reference standards of two cannabinoids, including delta-9-tetrahydrocannabinol (Δ9-THC) and cannabidiol (CBD), were purchased from Cayman Chemical, Ann Arbor, MI, USA. Ten microliters of each standard solution (1 mg/mL in methanol) were mixed with 90 μL of analytical grade methanol to constitute 100 µg/mL working solutions. The working solutions were stored at −20 °C.

#### 4.2.3. HPTLC Profiling

Twenty milligrams of ground *Cannabis* samples underwent extraction with 1 mL of analytical-grade dichloromethane (Honeywell, Offenbach, Germany) through a 30-min sonication process. Analysis of all samples was conducted using HPTLC Silica gel 60 F254 20 × 10 cm plates (Sigma-Aldrich, Oakville, ON, Canada). An 8-mm band on the HPTLC plates received three microliters of each cannabinoid standard and the analyte solutions, applied by a CAMAG automatic TLC Sampler (CAMAG Scientific Inc., Wilmington, NC, USA). After 15 min of humidity control and tank saturation, standards and samples were developed using a diethylamine-toluene (6:94) mobile phase system for 30 min. CAMAG TLC visualizer facilitated the visualization and imaging of the developed plates, with the solvent front approximately 70 mm for all plates. Derivatization was achieved by spraying 0.2% Fast Blue Salt B onto the developed plates using CAMAG. The documentation and analysis of the HPTLC profiling results were carried out using VisionCATS CAMAG HPTLC software (version 3.2, CAMAG Scientific Inc., Wilmington, NC, USA).

#### 4.2.4. UPLC Quantitative Analysis of THC and CBD

The quantitative analysis of THC and CBD was conducted by UPLC using Agilent 1290 Infinity II (Agilent Technologies, Santa Clara, CA, USA) equipped with an Agilent ZORBAX Eclipse Plus C18 (2.1 × 50 mm (1.8 um) (Agilent Technologies, Santa Clara, CA, USA). The analytical column was maintained at 40 °C with an injection volume of 2 µL and a flow rate of 0.5 mL/min. The chromatographic separation was achieved using a 6.0 min isocratic elution of 15% (A) 0.2% formic acid in water and 85% (B) 0.2% formic acid in methanol. Formic acid was purchased from Sigma-Aldrich, Saint Louis, MO, USA. Detection was carried out at 210 nm for preparation of standards, THC and CBD were dissolved in methanol to obtain a concentration range of 3.125–100 µg/mL Representative UPLC chromatograms and the standard curves of THC and CBD were shown in the Supplementary Information (Figure S1).

### 4.3. Development of DNA Markers Based on THCAS and CBDAS

#### 4.3.1. DNA Extraction

Fresh leaves are placed in a paper envelope and buried in a container filled with silica gel desiccant to dry the cannabis samples. The extraction of whole genomic DNA from each cultivar involved processing 20 mg of dried leaves using a Qiagen DNeasy Plant Mini Kit (QIAGEN, Toronto, ON, Canada). DNA concentrations were quantified using a NanoDrop^TM^ Microvolume UV-Vis Spectrophotometer (Thermo Fisher Scientific, Waltham, MA, USA).

#### 4.3.2. Primer Design

Primers for PCR amplification of full-length coding *THCAS* and *CBDAS* were designed following the previous methodology [35] (Table S2). Primers for *Cannabis* type prediction through DNA analysis were designed for two pairs of drug-type specific *THCAS* and two pairs of fiber-type specific *CBDAS*. Both sets of primers for prediction have their respective internal standards, as listed in Table S2.

#### 4.3.3. PCR Amplification

The total reaction volume for each reaction was 10 µL, comprising 1X Platinum^TM^ II PCR Buffer, 0.2 mM dNTPs, 0.2 µM target primers, 0.1 µM internal primers, 0.04 U/µL Platinum^TM^ II Taq Hot-Start DNA Polymerase, and 1 µL DNA template. All PCR reagents were procured from Thermo Fisher Scientific, Waltham, MA, USA. The PCR conditions consisted of pre-denaturation at 94 °C for 2 min, followed by 35 cycles of denaturation at 94 °C for 15 s, annealing at 64 °C for 15 s, and extension at 68 °C for 8 s. Subsequently, the PCR products were separated on 2% agarose gels and visualized under UV light (254 nm).

### 4.4. Data Analysis

#### 4.4.1. Heat Map Analysis of Chemical Profile

Data from UPLC analysis of cannabinoids chemical profiles, including Δ9-THC and CBD, was clustered by heatmap using the Euclidean clustering metric and Complete method of the pheatmap package in RStudio (Version 1.4.1103, Posit PBC, Boston, MA, USA.

#### 4.4.2. Phylogenetic Tree

Phylogenetic analysis of DNA sequencing data for *THCAS* and *CBDAS* was conducted using the CIPRES Science Gateway (https://www.phylo.org/) accessed on 31 May 2024. The accession numbers of reference sources and *Cannabis* samples were provided in Supplementary Table S1. Sequences were aligned using MAFFT on XSEDE [43]. The substitution model selected was TIM1+G for *THCAS* and *CBDAS*, and TIM1+I for consensus *THCAS*+*CBDAS*, with the number of substitutions set at eleven, determined using Jmodeltest2 on XSEDE (2.1.10) [44]. Maximum Likelihood analysis was performed on XSEDE using IQ-Tree on ACCESS (2.2.2.7) [45] with 1000 bootstrap replicates.

## Figures and Tables

**Table 1:** Summary results of Chemotype and Genotype of 85 samples of 46 *Cannabis* cultivars. The accuracy percentage of Chemotype and Genotype matching is 100% (85/85). Red, blue, and green text indicated Chemotype I, Chemotype II, and Chemotype III, respectively.

Sample	Chemotype				Matching Between Genotype & Chemotype	Genotype		
	%THC	%CBD	Log %THC /%CBD	Results		Results	*THCAS*	*CBDAS*
							1349 G/T	645 C/G
TK1	0.744	2.823	−0.579	**Chemotype II**	**Y**	**TD**	T	D
TK2	0.928	0.062	1.175	**Chemotype I**	**Y**	**Td**	T	d
TK3	1.657	0.155	1.029	**Chemotype I**	**Y**	**Td**	T	d
TK4	2.084	0.144	1.159	**Chemotype I**	**Y**	**Td**	T	d
TK6	0.831	0.03	1.437	**Chemotype I**	**Y**	**Td**	T	d
TK7	1.8	0.138	1.114	**Chemotype I**	**Y**	**Td**	T	d
TK8	0.953	0.077	1.092	**Chemotype I**	**Y**	**Td**	T	d
TK9	1.968	0.077	1.405	**Chemotype I**	**Y**	**Td**	T	d
TK11	1.045	0.141	0.869	**Chemotype I**	**Y**	**Td**	T	d
TK12	0.503	0.034	1.172	**Chemotype I**	**Y**	**Td**	T	d
TK13	1.547	0.12	1.111	**Chemotype I**	**Y**	**Td**	T	d
TK14	1.339	3.053	−0.358	**Chemotype II**	**Y**	**TD**	T	D
TK17	0.614	0.035	1.249	**Chemotype I**	**Y**	**Td**	T	d
TK18	1.472	0.14	1.023	**Chemotype I**	**Y**	**Td**	T	d
TK19	1.212	0.024	1.709	**Chemotype I**	**Y**	**Td**	T	d
TK20	2.174	0.12	1.26	**Chemotype I**	**Y**	**Td**	T	d
TK25	2.052	0.116	1.249	**Chemotype I**	**Y**	**Td**	T	d
TK28	1.21	0.097	1.095	**Chemotype I**	**Y**	**Td**	T	d
TK29	1.656	0.08	1.313	**Chemotype I**	**Y**	**Td**	T	d
TK33	1.144	0.03	1.574	**Chemotype I**	**Y**	**Td**	T	d
TK34	1.317	0.043	1.489	**Chemotype I**	**Y**	**Td**	T	d
TK36	2.183	0.053	1.615	**Chemotype I**	**Y**	**Td**	T	d
TK37	1.688	0.106	1.203	**Chemotype I**	**Y**	**Td**	T	d
TK40	0.85	0.048	1.249	**Chemotype I**	**Y**	**Td**	T	d
TK41	1.195	0.023	1.722	**Chemotype I**	**Y**	**Td**	T	d
TK42	1.015	0.014	1.855	**Chemotype I**	**Y**	**Td**	T	d
TK43	1.72	0.048	1.55	**Chemotype I**	**Y**	**Td**	T	d
TK44	1.885	0.044	1.635	**Chemotype I**	**Y**	**Td**	T	d
TK45	0.538	1.341	−0.397	**Chemotype II**	**Y**	**TD**	T	D
TK46	1.884	0.062	1.482	**Chemotype I**	**Y**	**Td**	T	d
TK47	0.445	0.008	1.723	**Chemotype I**	**Y**	**Td**	T	d
TK48	0.374	0.011	1.547	**Chemotype I**	**Y**	**Td**	T	d
TK51	1.907	0.051	1.572	**Chemotype I**	**Y**	**Td**	T	d
TK52	0.488	0.002	2.301	**Chemotype I**	**Y**	**Td**	T	d
TK55	1.638	0.037	1.65	**Chemotype I**	**Y**	**Td**	T	d
TK56	2.475	0.056	1.649	**Chemotype I**	**Y**	**Td**	T	d
TK57	1.518	0.034	1.646	**Chemotype I**	**Y**	**Td**	T	d
TK58	0.782	0.018	1.632	**Chemotype I**	**Y**	**Td**	T	d
TK59	0.913	0.035	1.415	**Chemotype I**	**Y**	**Td**	T	d
TK60	0.065	2.382	−1.561	**Chemotype III**	**Y**	**tD**	t	D
TK61	0.072	2.96	−1.613	**Chemotype III**	**Y**	**tD**	t	D
TK62	0.784	0.039	1.306	**Chemotype I**	**Y**	**Td**	T	d
TK63	0.852	0.01	1.921	**Chemotype I**	**Y**	**Td**	T	d
TK64	0.838	0.041	1.308	**Chemotype I**	**Y**	**Td**	T	d
TK65	1.394	0.001	3.144	**Chemotype I**	**Y**	**Td**	T	d
TK69	0.892	0.001	2.95	**Chemotype I**	**Y**	**Td**	T	d
TK70	0.48	0.001	2.682	**Chemotype I**	**Y**	**Td**	T	d
TK73	1.251	0.029	1.641	**Chemotype I**	**Y**	**Td**	T	d
TK74	0.468	0.001	2.67	**Chemotype I**	**Y**	**Td**	T	d
TK76	1.66	0.038	1.637	**Chemotype I**	**Y**	**Td**	T	d
TK77	2.036	0.052	1.591	**Chemotype I**	**Y**	**Td**	T	d
TK78	0.931	0.001	2.969	**Chemotype I**	**Y**	**Td**	T	d
TK86	0.817	0.042	1.289	**Chemotype I**	**Y**	**Td**	T	d
TK88	0.209	0.001	2.32	**Chemotype I**	**Y**	**Td**	T	d
TK89	0.611	0.001	2.786	**Chemotype I**	**Y**	**Td**	T	d
TK90	0.977	0.033	1.468	**Chemotype I**	**Y**	**Td**	T	d
TK92	2.86	0.069	1.616	**Chemotype I**	**Y**	**Td**	T	d
TK94	0.712	0.001	2.853	**Chemotype I**	**Y**	**Td**	T	d
TK96	0.874	0.032	1.441	**Chemotype I**	**Y**	**Td**	T	d
TK97	0.542	1.309	−0.383	**Chemotype II**	**Y**	**TD**	T	D
TK98	0.536	1.332	−0.396	**Chemotype II**	**Y**	**TD**	T	D
TK101	0.306	0.015	1.322	**Chemotype I**	**Y**	**Td**	T	d
TK102	0.906	0.056	1.212	**Chemotype I**	**Y**	**Td**	T	d
TK103	0.071	0.861	−1.085	**Chemotype III**	**Y**	**tD**	t	D
TK105	0.847	0.023	1.564	**Chemotype I**	**Y**	**Td**	T	d
TK106	0.878	0.017	1.717	**Chemotype I**	**Y**	**Td**	T	d
TK108	0.499	0.002	2.311	**Chemotype I**	**Y**	**Td**	T	d
TK111	2.144	0.132	1.21	**Chemotype I**	**Y**	**Td**	T	d
TK115	0.881	0.025	1.554	**Chemotype I**	**Y**	**Td**	T	d
TK117	0.107	0.018	0.762	**Chemotype I**	**Y**	**Td**	T	d
TK118	0.92	0.015	1.795	**Chemotype I**	**Y**	**Td**	T	d
TK121	1.107	0.016	1.828	**Chemotype I**	**Y**	**Td**	T	d
TK122	1.178	0.055	1.33	**Chemotype I**	**Y**	**Td**	T	d
TK126	1.517	3.371	−0.347	**Chemotype II**	**Y**	**TD**	T	D
TK127	1.695	3.039	−0.254	**Chemotype II**	**Y**	**TD**	T	D
TK132	3.678	4.598	−0.097	**Chemotype II**	**Y**	**TD**	T	D
TK133	2.244	0.068	1.518	**Chemotype I**	**Y**	**Td**	T	d
TK134	3.122	0.1	1.494	**Chemotype I**	**Y**	**Td**	T	d
TK136	1.179	0.043	1.435	**Chemotype I**	**Y**	**Td**	T	d
TK137	2.028	0.078	1.413	**Chemotype I**	**Y**	**Td**	T	d
TK138	2.796	0.143	1.292	**Chemotype I**	**Y**	**Td**	T	d
TK139	0.141	2.776	−1.294	**Chemotype III**	**Y**	**tD**	t	D
TK140	2.676	6.495	−0.385	**Chemotype II**	**Y**	**TD**	T	D
TK61F1-C0-2G	1.381	3.388	−0.39	**Chemotype II**	**Y**	**TD**	T	D
TK61F1-C0-5P	0.507	1.658	−0.515	**Chemotype II**	**Y**	**TD**	T	D

## Data Availability

The original DNA sequencing data presented in the study are openly available in DNA Data Bank of Japan at https://ddbj.nig.ac.jp/arsa. All accession numbers were listed in Table S1.

## Supplementary Materials

The following supporting information can be downloaded at: https://www.mdpi.com/article/10.3390/ijms26115077/s1 [46,47,48,49].
