# Supplementary material for: Rapid Specific PCR Detection Based on THCAS and CBDAS for the Prediction of Cannabis sativa Chemotypes: Drug, Fiber, and Intermediate
Source: Int J Mol Sci. 2025 May 24;26(11):5077. doi: 10.3390/ijms26115077 (PMC12154019; doi:10.3390/ijms26115077)
Supplement: Supplementary file 1 [file ijms-26-05077-s001.zip › Figure S1.pdf]

A

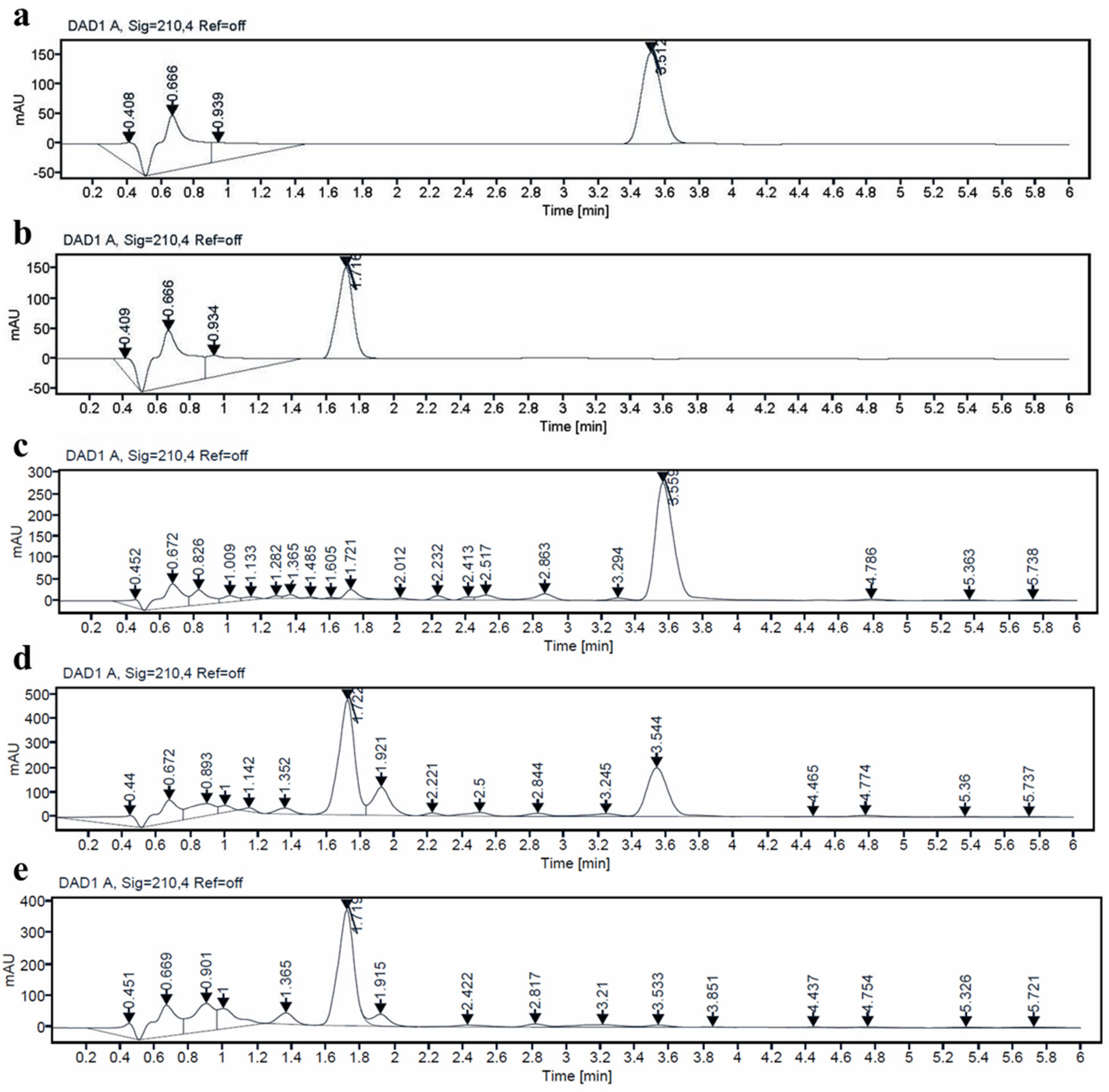
a
DAD1 A, Sig=210,4 Ref=off
mAU
Time [min]
0.408
0.666
0.939
3.512
b
DAD1 A, Sig=210,4 Ref=off
mAU
Time [min]
0.409
0.666
0.934
1.718
c
DAD1 A, Sig=210,4 Ref=off
mAU
Time [min]
0.452
0.672
0.826
1.009
1.133
1.282
1.365
1.485
1.605
1.721
2.012
2.232
2.413
2.517
2.863
3.294
3.559
4.786
5.363
5.738
d
DAD1 A, Sig=210,4 Ref=off
mAU
Time [min]
0.44
0.672
0.893
1
1.142
1.352
1.722
1.921
2.221
2.5
2.844
3.245
3.544
4.465
4.774
5.36
5.737
e
DAD1 A, Sig=210,4 Ref=off
mAU
Time [min]
0.451
0.669
0.901
1
1.365
1.719
1.915
2.422
2.817
3.21
3.533
3.851
4.437
4.754
5.326
5.721

B

a

b

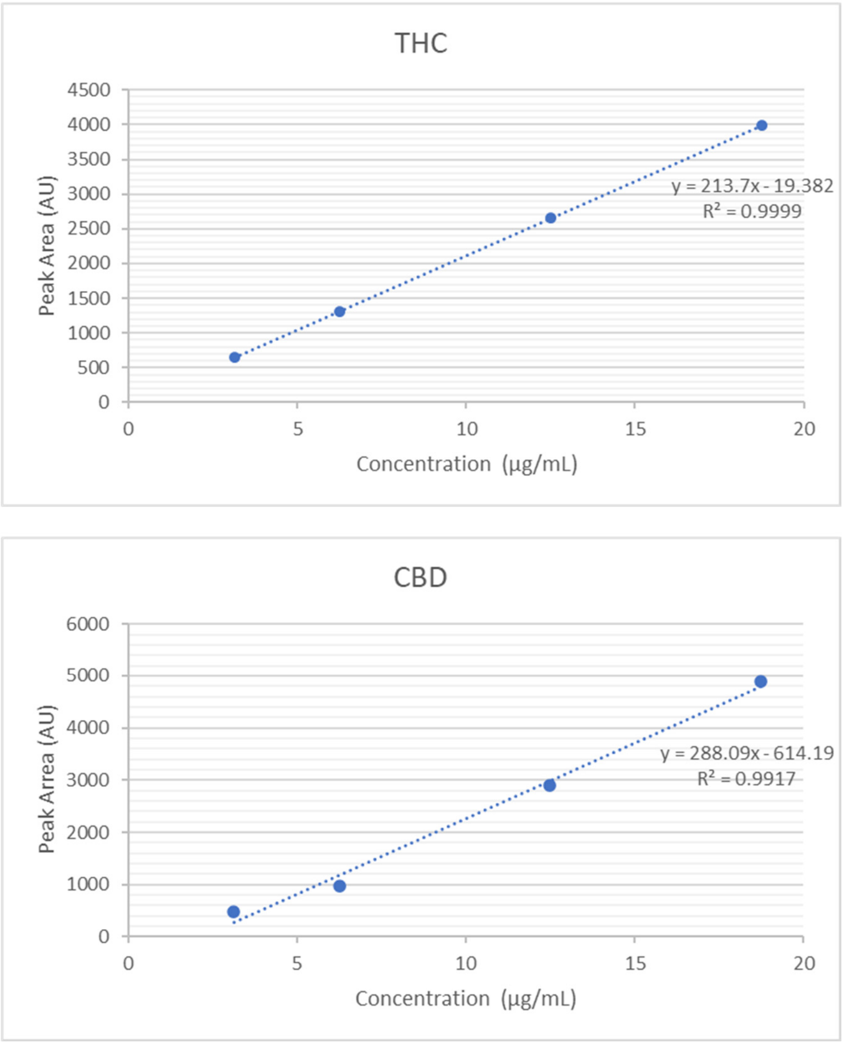

Figure S1: UPLC Chromatogram and Standard Curve **(A)** UPLC Chromatogram for (a) THC Standard, (b) CBD Standard, **(c)** Drug-type sample, **(d)** Intermediate-type sample, and **(e)** Fiber-type sample and **(B)** UPLC Standard Curve for (a) THC and (b) CBD.
