# Supplementary material for: Rapid Specific PCR Detection Based on THCAS and CBDAS for the Prediction of Cannabis sativa Chemotypes: Drug, Fiber, and Intermediate
Source: Int J Mol Sci. 2025 May 24;26(11):5077. doi: 10.3390/ijms26115077 (PMC12154019; doi:10.3390/ijms26115077)
Supplement: Supplementary file 1 [file ijms-26-05077-s001.zip › Figure S2.pdf]

A *THCAS* TK2 (Drug-type)

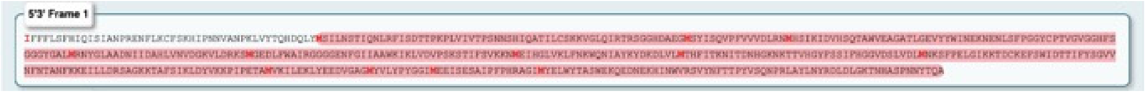

THCAS TK20 (Drug-type)

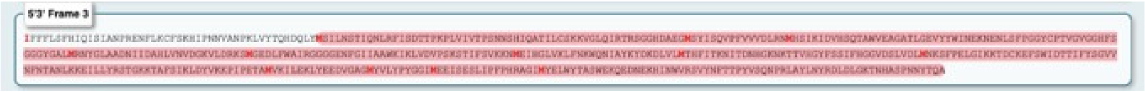

THCAS TK55 (Drug-type)

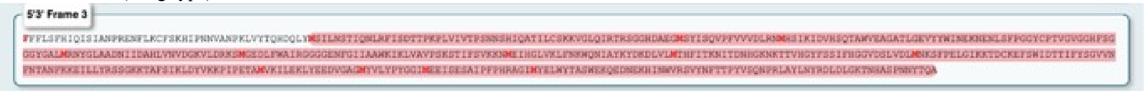

THCAS TK137 (Drug-type)

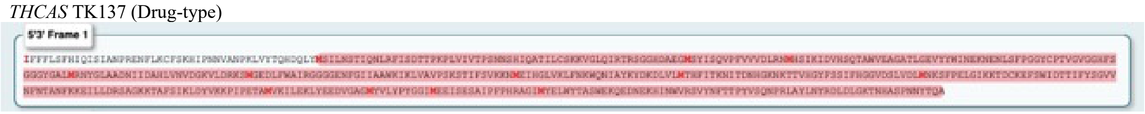

THCAS TK1 (Intermediate-type)

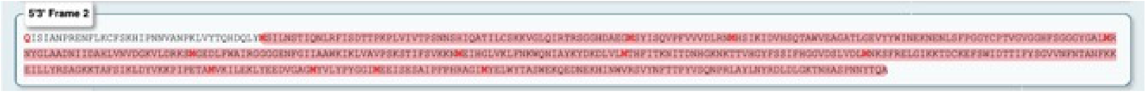

THCAS TK97 (Intermediate-type)

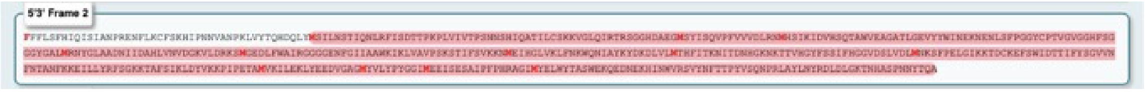

THCAS TK127 (Intermediate-type)

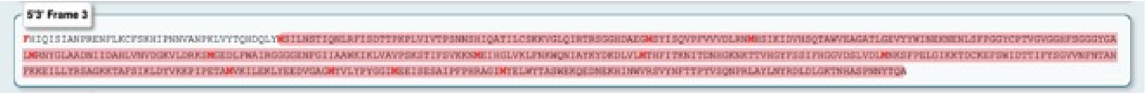

THCAS TK61F1-CO-2G (Intermediate-type)

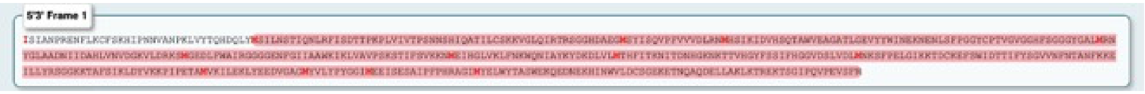

THCAS TK61F1-CO-5P (Intermediate-type)

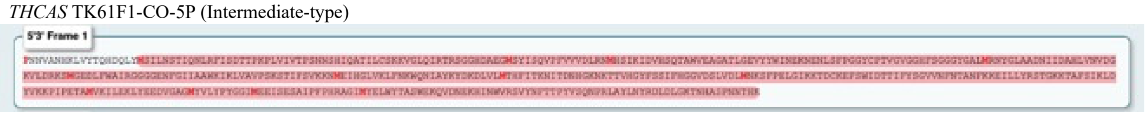

THCAS TK60 (Fiber-type)

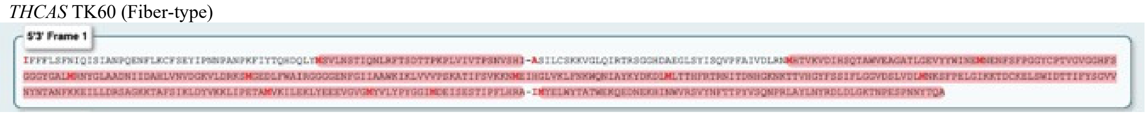

THCAS TK61 (Fiber-type)

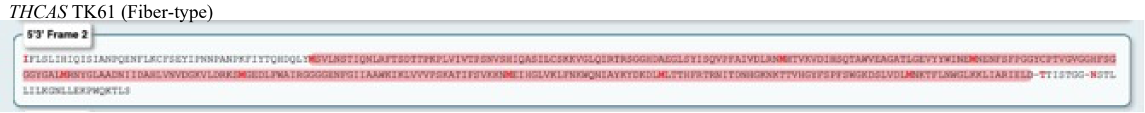

THCAS TK139 (Fiber-type)

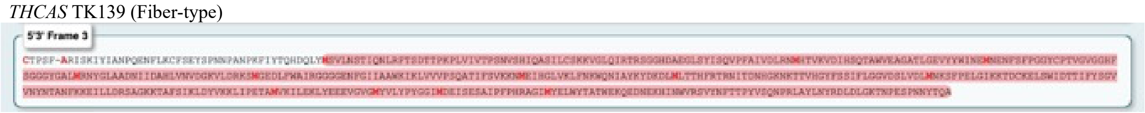

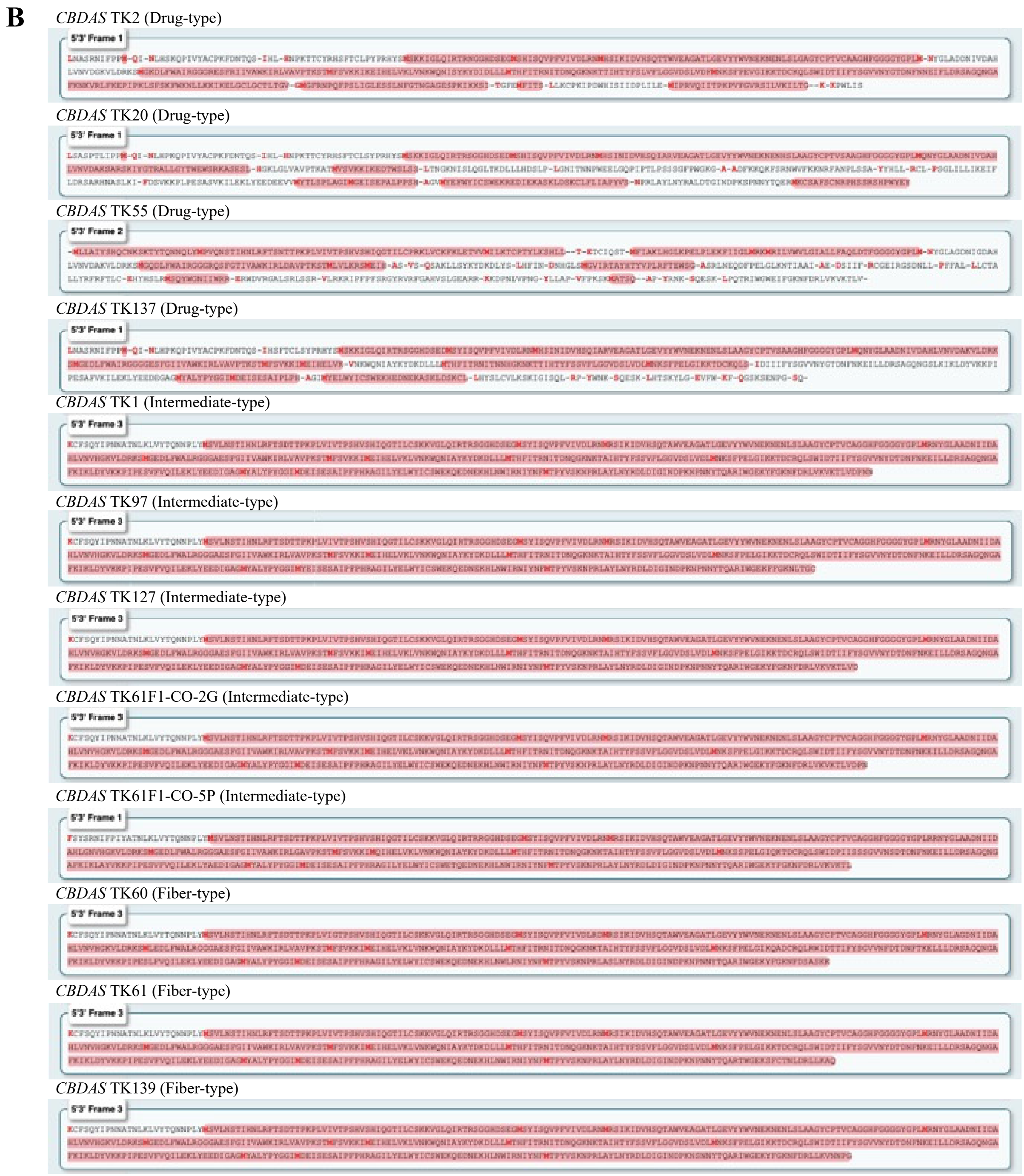

Figure S2: Amino acid sequence of **(A)** *THCAS* and **(B)** *CBDAS* including TK2, TK20, TK55, and TK137 (Drug-type), TK1, TK97, TK127, TK61F1-CO-2G and TK61F1-CO-5P (Intermediate-type), TK60, TK61, and TK139 (Fiber-type).
