# Supplementary material for: Rapid Specific PCR Detection Based on THCAS and CBDAS for the Prediction of Cannabis sativa Chemotypes: Drug, Fiber, and Intermediate
Source: Int J Mol Sci. 2025 May 24;26(11):5077. doi: 10.3390/ijms26115077 (PMC12154019; doi:10.3390/ijms26115077)
Supplement: Supplementary file 1 [file ijms-26-05077-s001.zip › Figure S4.pdf]

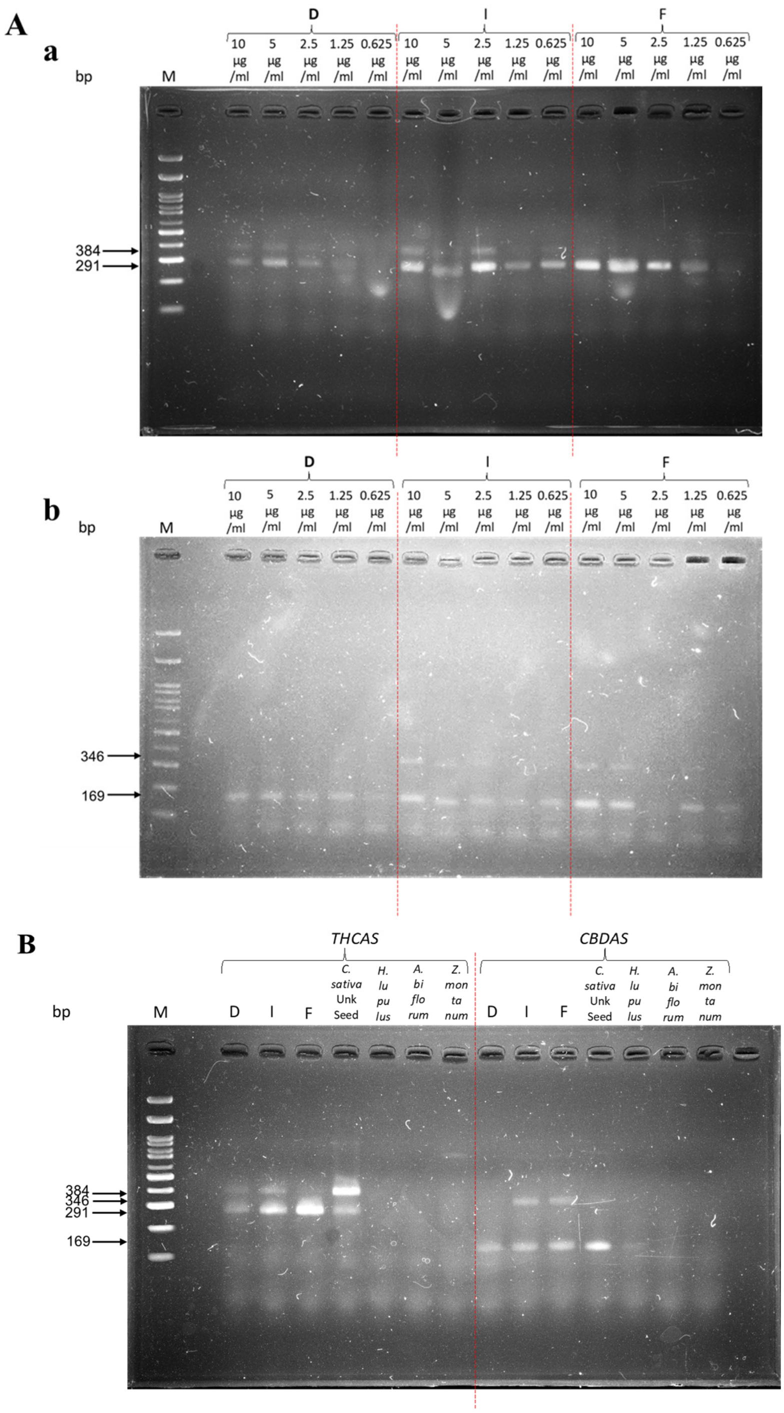

Figure S4: PCR amplicons for *THCAS* and *CBDAS*. **(A)** Sensitivity test, DNA was extracted from 3 representative cultivars—TK19 (Drug-type), TK97 (Intermediate-type), and TK60 (Fiber-type)—and two-fold serial diluted from 10 µg/ml to 0.625 µg/ml; **(a)** *THCAS,* Expected PCR products for *THCAS*d (384 bp) with *THCAS*int (291 bp) internal control; **(b)** *CBDAS,* Expected PCR products for *CBDAS*f (346 bp) with *CBDAS*int (169 bp) internal control. **(B)** *THCAS* and *CBDAS* Specificity test, three representative cultivars—TK19 (Drug-type), TK97 (Intermediate-type), and TK60 (Fiber-type) were tested together with unidentified *Cannabis* seed, *Humulus lupulus*, *Amomum biflorum*, and *Zingiber montanum*.
