# Supplementary material for: Rapid Specific PCR Detection Based on THCAS and CBDAS for the Prediction of Cannabis sativa Chemotypes: Drug, Fiber, and Intermediate
Source: Int J Mol Sci. 2025 May 24;26(11):5077. doi: 10.3390/ijms26115077 (PMC12154019; doi:10.3390/ijms26115077)
Supplement: Supplementary file 1 [file ijms-26-05077-s001.zip › Figure S5.pdf]

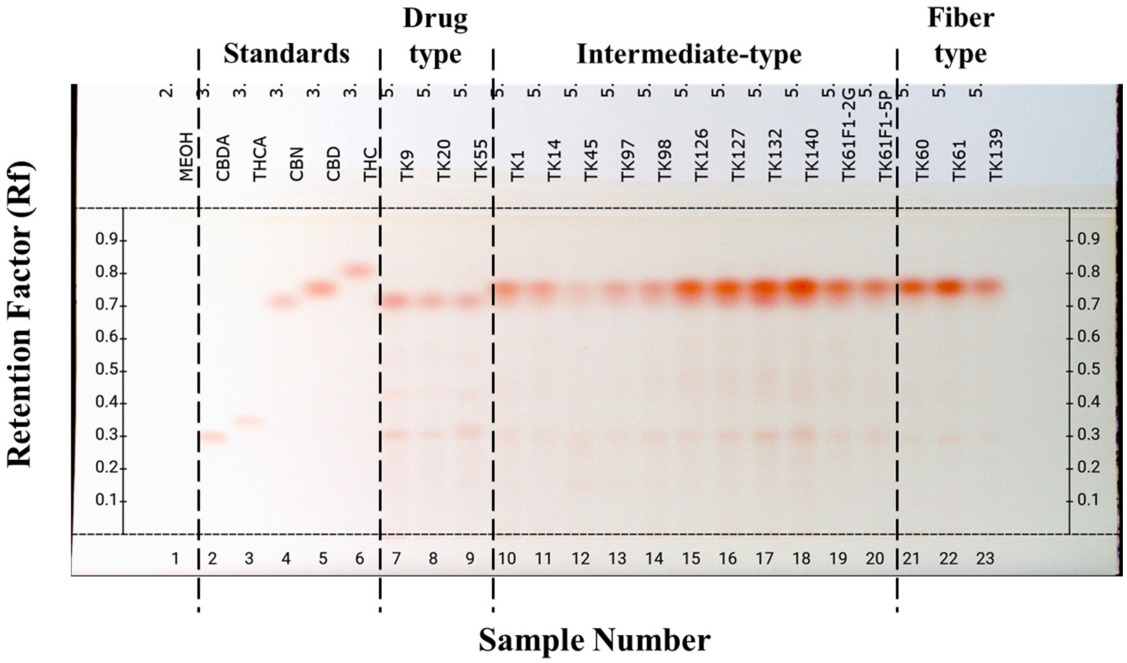

Figure S5: HPTLC chemical profile of 17 *Cannabis* samples on Hexane: Acetone 30: 10 mobile system. X-axis represented sample number. Y-axis represented R_F value. Lane 1: methanol blank; Lane 2-6: cannabinoid standards including CBDA, THCA, CBN, CBD, and THC; Lane 7-9: Drug-type sample including TK9, TK20, and TK55; Lane 10-20: Intermediate-type sample including TK1, TK14, TK45, TK97, TK98, TK126, TK127, TK132, TK140, TK61F1-C0-2G, and TK61F1-C0-5P; Lane 21-23: Fiber-type including TK60, TK61, and TK139.
