# Supplementary material for: Rapid Specific PCR Detection Based on THCAS and CBDAS for the Prediction of Cannabis sativa Chemotypes: Drug, Fiber, and Intermediate
Source: Int J Mol Sci. 2025 May 24;26(11):5077. doi: 10.3390/ijms26115077 (PMC12154019; doi:10.3390/ijms26115077)
Supplement: Supplementary file 1 [file ijms-26-05077-s001.zip › Figure S6.pdf]

Title	**A Rapid Method for Drug-type, Fiber-type, and Intermediate *Cannabis* Plant Differentiation using Chemotypes and *THCAS* and *CBDAS* DNA Markers**
Authors	Patwira Boonjing[1,2], Worakorn Wiwatcharakornkul[2], Chayapol Tungphatthong[1], Wanchai De-Eknamkul[2,3], Somchai Keawwangchai[4], Tae-Jin Yang[5] & Suchada Sukrong[1,2*]
Affiliation	[1]Center of Excellence in DNA Barcoding of Thai Medicinal Plants, Chulalongkorn University, Bangkok 10330, Thailand.
	[2] Department of Pharmacognosy and Pharmaceutical Botany, Faculty of Pharmaceutical Sciences, Chulalongkorn University, Bangkok 10330, Thailand
	[3] Natural Product Biotechnology Research Unit, Chulalongkorn University, Bangkok 10330, Thailand
	[4]Department of Chemistry, Faculty of Science, Mahasarakham University, Mahasarakham 44150, Thailand
	[5]Department of Agriculture, Forestry and Bioresources, Plant Genomics & Breeding Institute, College of Agriculture & Life Sciences, Seoul National University, 1 Gwanak-ro, Gwanak-gu, Seoul 08826, Korea
*Corresponding author	Professor Suchada Sukrong, Ph.D.
	Research Unit of DNA Barcoding of Thai Medicinal Plants,
	Department of Pharmacognosy and Pharmaceutical Botany,
	Faculty of Pharmaceutical Sciences, Chulalongkorn University,
	Bangkok 10330, Thailand
	Phone: +6681-819-6742, Fax: +6622-558-227
	Email: suchada.su@chula.ac.th

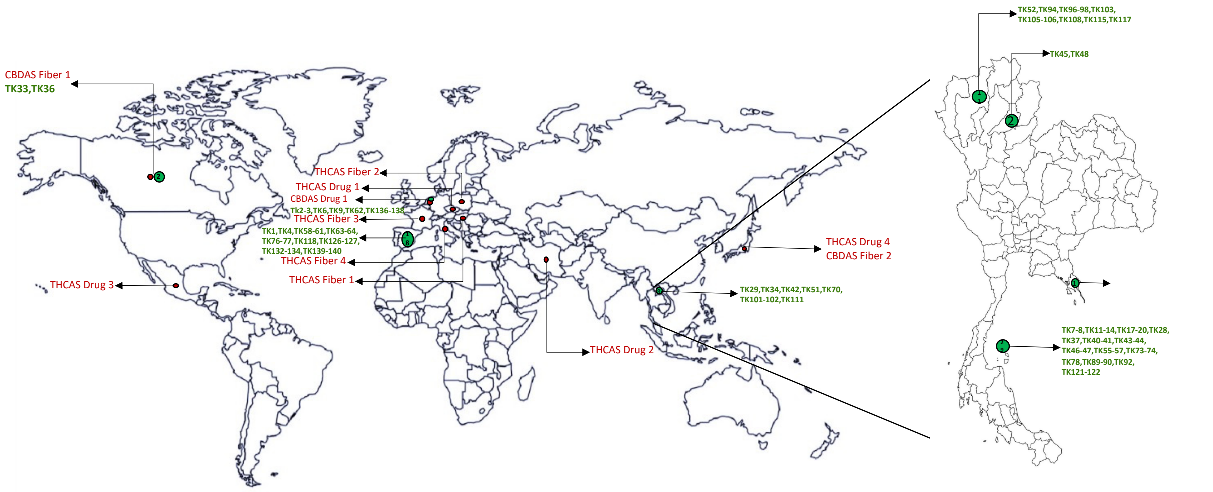

Figure S6: *Cannabis sativa* sample locations in this study. Green points indicated Thai *Cannabis* samples collected in this study. Red points indicated samples from references as mentioned in Table S1.
