# Supplementary material for: Rapid Specific PCR Detection Based on THCAS and CBDAS for the Prediction of Cannabis sativa Chemotypes: Drug, Fiber, and Intermediate
Source: Int J Mol Sci. 2025 May 24;26(11):5077. doi: 10.3390/ijms26115077 (PMC12154019; doi:10.3390/ijms26115077)
Supplement: Supplementary file 1 [file ijms-26-05077-s001.zip › Table S1.pdf]

Table S1: List of *Cannabis sativa* samples collection details (no.1-85) and references (no.86-99) including ID, cultivar name, location of origin, voucher number, and GenBank accession number (GenBank accession number of references received from [22] (no.86-93), [46] (no.94-95), [47] (no.96), [48] (no.97 and no.99), and [49] (no.98)).

No.	ID	Cultivar name	Location of Origin	Voucher no.	GenBank Accession no.	
					THCAS	***CBDAS***
1	TK1	Silver Lime Haze	Spain	SS-898	LC807849	LC807861
2	TK2	Royal Queen Dimond Auto	The Netherlands	SS-899	LC807845	LC807856
3	TK3	Skunk Medical .1	The Netherlands	SS-900	-	-
4	TK4	Amnesi K lemon fem	Spain	SS-901	-	-
5	TK6	Skunk Medical .2	The Netherlands	SS-902	-	-
6	TK7	Thai KD STS .1	Surat Thani, Thailand	SS-903	-	-
7	TK8	Thai KD STS .2	Surat Thani, Thailand	SS-904	-	-
8	TK9	Skunk Medical .3	The Netherlands	SS-905	-	-
9	TK11	KD 1.2	Surat Thani, Thailand	SS-906	-	-
10	TK12	KD 1.3	Surat Thani, Thailand	SS-907	-	-
11	TK13	Dabphasutha Muangmao .1	Surat Thani, Thailand	SS-908	-	-
12	TK14	Prasobsuk 3	Surat Thani, Thailand	SS-909	-	-
13	TK17	KD 2.1	Surat Thani, Thailand	SS-910	-	-
14	TK18	Inthanin .2	Surat Thani, Thailand	SS-911	-	-
15	TK19	GBT KD STS .1	Surat Thani, Thailand	SS-912	-	-
16	TK20	KD 2.2	Surat Thani, Thailand	SS-913	LC807846	LC807858
17	TK25	Big Head	Unknown	SS-914	-	-
18	TK28	Inthanin .3	Surat Thani, Thailand	SS-915	-	-
19	TK29	Blue OG+Mango .2	Laos	SS-916	-	-
20	TK33	Y2K .3	United States of America	SS-917	-	-
21	TK34	Blue OG+Mango .3	Laos	SS-918	-	-
22	TK36	Y2K .4	United States of America	SS-919	-	-
23	TK37	KD 3 .1	Surat Thani, Thailand	SS-920	-	-
24	TK40	Thai KD STS .3	Surat Thani, Thailand	SS-921	-	-
25	TK41	Thai KD STS .4	Surat Thani, Thailand	SS-922	-	-
26	TK42	Mango Thai F4 .4	Laos	SS-923	-	-
27	TK43	GBT KD STS .2	Surat Thani, Thailand	SS-924	-	-
28	TK44	GBT KD STS .3	Surat Thani, Thailand	SS-925	-	-
29	TK45	Maelua .1	Phrae, Thailand	SS-926	-	-
30	TK46	Thaweesuk .2	Surat Thani, Thailand	SS-927	-	-

No.	ID	Cultivar name	Location of Origin	Voucher no.	GenBank Accession no.	
					THCAS	*CBDAS*
31	TK47	Thaweesuk .3	Surat Thani, Thailand	SS-928	-	-
32	TK48	Maelua .2	Phrae, Thailand	SS-929	-	-
33	TK51	Blue OG+Mango .4	Laos	SS-930	-	-
34	TK52	CMX Samerng	Chiang Mai, Thailand	SS-931		
35	TK55	Inthanin .4	Surat Thani, Thailand	SS-932	LC807847	LC807859
36	TK56	KD 3 .3	Surat Thani, Thailand	SS-933	-	-
37	TK57	Chalasin .2	Surat Thani, Thailand	SS-934	-	-
38	TK58	White widow Auto	Spain	SS-935	-	-
39	TK59	AK 49 Auto	Spain	SS-936	-	-
40	TK60	Auto Pink Kusk CBD .1	Spain	SS-937	LC813052	LC807870
41	TK61	Auto Pink Kusk CBD .2	Spain	SS-938	LC813053	LC807871
42	TK62	Kermatic Auto	The Netherlands	SS-941	-	-
43	TK63	Royal Gorila .1	Spain	SS-942	-	-
44	TK64	Royal Gorila .2	Spain	SS-943	-	-
45	TK65	Super Lemon Mango .1	Unknown	SS-944	-	-
46	TK69	OG NYC .3	Unknown	SS-945	-	-
47	TK70	Mango OG	Laos	SS-946	-	-
48	TK73	Daengchakkrapad .1	Surat Thani, Thailand	SS-947	-	-
49	TK74	Daengchakkrapad .2	Surat Thani, Thailand	SS-948	-	-
50	TK76	Pie apple express .1	Spain	SS-949	-	-
51	TK77	Pie apple express .2	Spain	SS-950	-	-
52	TK78	Manee Theva	Surat Thani, Thailand	SS-951	-	-
53	TK86	Bom .3	Unknown	SS-952	-	-
54	TK88	Wild Thailand	Trat, Thailand	SS-953	-	-
55	TK89	KD .1	Surat Thani, Thailand	SS-954	-	-
56	TK90	KD .2	Surat Thani, Thailand	SS-955	-	-
57	TK92	KD .4	Surat Thani, Thailand	SS-956	-	-
58	TK94	Super Thai Skunk .1	Chiang Mai, Thailand	SS-957	-	-
59	TK96	Thai Sativa+OG Kush .1	Chiang Mai, Thailand	SS-958	-	-
60	TK97	Thai Sativa+OG Kush .2	Chiang Mai, Thailand	SS-959	LC807851	LC807863
61	TK98	Thai Sativa+OG Kush .3	Chiang Mai, Thailand	SS-960	-	-
62	TK101	Estella .2	Laos	SS-961	-	-
63	TK102	Estella .3	Laos	SS-962	-	-
64	TK103	Sativa Changkian .1	Chiang Mai, Thailand	SS-963	-	-
65	TK105	RPG Thai .1	Chiang Mai, Thailand	SS-964	-	-
66	TK106	RPG Thai .2	Chiang Mai, Thailand	SS-965	-	-
**No.**	**ID**	**Cultivar name**	**Location of Origin**		**GenBank Accession no.**	

				**Voucher no.**	***THCAS***	***CBDAS***
67	TK108	RPG Thai .4	Chiang Mai, Thailand	SS-966	-	-
68	TK111	Blue OG .1	Laos	SS-967	-	-
69	TK115	Thai Mango F2 .1	Chiang Mai, Thailand	SS-968	-	-
70	TK117	Thai Mango F2 .3	Chiang Mai, Thailand	SS-969	-	-
71	TK118	Gorrila .1	Spain	SS-970	-	-
72	TK121	GBT KD STS .4	Surat Thani, Thailand	SS-971	-	-
73	TK122	GBT KD STS .5	Surat Thani, Thailand	SS-972	-	-
74	TK126	Royal medics	Spain	SS-973	-	-
75	TK127	Royal medics	Spain	SS-974	LC807852	LC807865
76	TK132	OG Kush CBD	Spain	SS-975	-	-
77	TK133	ACDC	Spain	SS-976	-	-
78	TK134	ACDC	Spain	SS-977	-	-
79	TK136	Buddha MEDICAL	The Netherlands	SS-978	-	-
80	TK137	Buddha MEDICAL	The Netherlands	SS-979	LC807848	LC807860
81	TK138	Buddha MEDICAL	The Netherlands	SS-980	-	-
82	TK139	Royal Highness	Spain	SS-981	LC807855	LC807873
83	TK140	Royal Highness	Spain	SS-982	-	-
84	TK61F1 -C0-2G	TK61 and Estella hybrid	Spain and Laos	SS-939	LC807853	LC807868
85	TK61F1 -C0-5P	TK61 and Estella hybrid	Spain and Laos	SS-940	LC807854	LC807869
86	*THCAS* Drug 1	strain:001	Czech	-	AB212829	-
87	*THCAS* Drug 2	strain:010	Iran	-	AB212832	-
88	*THCAS* Drug 3	strain:013	Mexico	-	AB212834	-
89	*THCAS* Drug 4	strain:053	Japan	-	AB212837	-
90	*THCAS* Fiber 1	strain:005	Hungary	-	AB212830	-
91	*THCAS* Fiber 2	strain:009	Poland	-	AB212831	-
92	*THCAS* Fiber 3	strain:066	France	-	AB212839	-
93	*THCAS* Fiber 4	strain:078	Italy	-	AB212841	-
94	*CBDAS* Drug 1	Skunk #1 homolog 2	The Netherlands	-	-	KJ469376
95	*CBDAS* Fiber 1	Carmen	Canada	-	-	KJ469374
96	*CBDAS* Fiber 2	CBDA strain	Japan	-	-	AB292682
**No.**	**ID**	**Cultivar name**	**Location of Origin**		**GenBank Accession no.**	

				Voucher no.	***THCAS***	***CBDAS***
97	*THCAS*-like	Like_Drug1	Italy	-	MW429551.1	-
98	*CBDAS*-like	-	-	-	-	NM001397936.1
99	*CBCAS*	Ab0	Italy	-	MW561076.1	-
