# Supplementary material for: Rapid Specific PCR Detection Based on THCAS and CBDAS for the Prediction of Cannabis sativa Chemotypes: Drug, Fiber, and Intermediate
Source: Int J Mol Sci. 2025 May 24;26(11):5077. doi: 10.3390/ijms26115077 (PMC12154019; doi:10.3390/ijms26115077)
Supplement: Supplementary file 1 [file Figure S3.pdf › Figure S3.pdf]

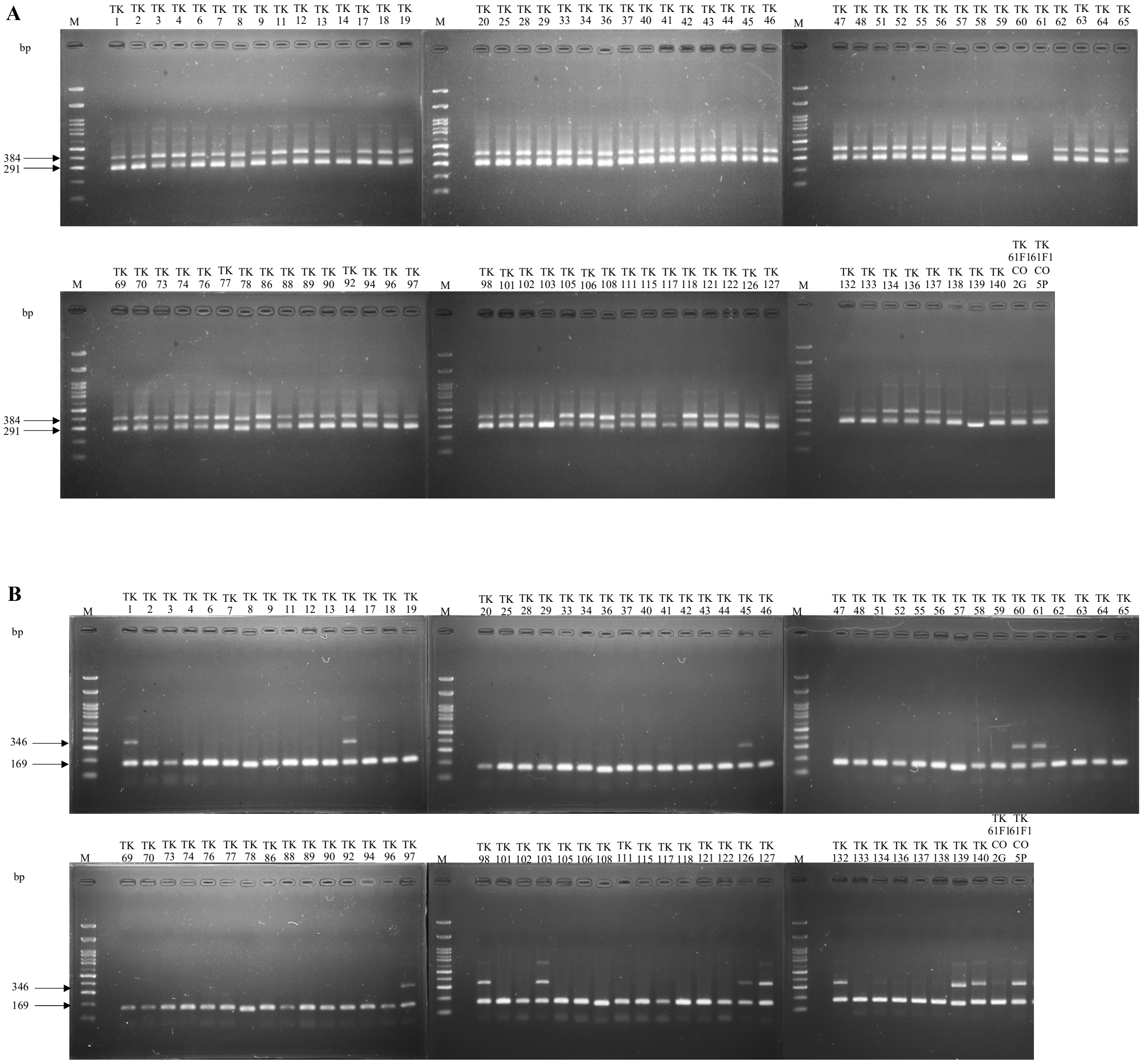

Figure S3: PCR amplicons for *THCAS* and *CBDAS.* **(A)** Primer pair D (*THCAS*d) with internal control pair C (*THCAS*int), and **(B)** Primer pair F (*CBDAS*f) with internal control pair E (*CBDAS*int) of 85 *Cannabis* cultivars.
